# Atorvastatin Attenuates Endothelial Cell Injury in Atherosclerosis Through Inhibiting ACSL4-Mediated Ferroptosis

**DOI:** 10.1155/2024/5522013

**Published:** 2024-06-20

**Authors:** Huilian Tan, Ling Liu, Yanchao Qi, Dahong Zhang, Yanchun Zhi, Yu Li, Huimin Zhang, Jun Liu

**Affiliations:** Department of Cardiology The First Hospital of Hebei Medical University, Shijiazhuang, Hebei, China

**Keywords:** ACSL4, atherosclerosis, atorvastatin, endothelial cell injury, ferroptosis

## Abstract

**Objective:** This study is aimed at investigating the effects of atorvastatin (ATV) on endothelial cell injury in atherosclerosis (AS) through inhibiting acyl-CoA synthetase long-chain family member 4 (ACSL4)-mediated ferroptosis.

**Methods:** Human umbilical vein endothelial cells (HUVECs) were treated with oxidized low-density lipoprotein (ox-LDL) to establish an in vitro model of AS. The cell viability, lactate dehydrogenase (LDH) release, apoptosis, and expression levels of apoptotic proteins were assessed. The levels of inflammatory factors and adhesion molecules were determined by ELISA and Western blot, respectively. Cellular iron content, lipid peroxidation, glutathione (GSH) levels, and lipid reactive oxygen species (ROS) were measured. ACSL4 overexpression was performed to investigate its role in ATV-mediated protection against ferroptosis.

**Results:** ATV alleviated ox-LDL-induced HUVEC damage by restoring cell viability, reducing LDH levels, and inhibiting apoptosis. It also attenuated inflammation and adhesion by decreasing the levels of inflammatory factors TNF-*α*, IL-6, and IL-8, as well as adhesion molecules ICAM-1 and VCAM-1. ATV inhibited ferroptosis by regulating iron content, malondialdehyde (MDA) levels, ROS levels, and ACSL4 protein expression. Overexpression of ACSL4 (oe-ACSL4) hindered the protective effects of ATV on cell viability, antiapoptotic protein expression, LDH levels, apoptosis, and inflammatory factors.

**Conclusion:** Our findings suggest that ATV attenuates endothelial cell injury in AS by inhibiting ACSL4-mediated ferroptosis. These results provide insights into the potential therapeutic strategies for the treatment of AS.

## 1. Introduction

Atherosclerosis (AS) is a disease characterized by the accumulation of fibrous plaques on the arterial walls, leading to the narrowing of blood vessels and potential obstruction. It is a diffuse arterial disorder associated with various arterial diseases such as peripheral artery disease and coronary heart disease [1]. Intracranial atherosclerotic disease (ICAD) is considered the most common cause of ischemic stroke worldwide [1] and is a major cause of cardiovascular morbidity and mortality. This disease involves a series of events, including endothelial cell injury, lipoprotein deposition, inflammatory reactions, and the formation of smooth muscle cell caps [2]. Endothelial cells are the inner layer cells of the vascular endothelium, covering the inner wall of blood vessels and playing a crucial role in maintaining vascular function and structure. During the process of AS, endothelial cells are influenced by various proinflammatory factors and oxidative stress, leading to the activation of inflammatory reactions in endothelial cells. Such inflammation can promote leukocyte adhesion and infiltration into the vessel wall, thereby exacerbating plaque formation [3–5]. Protecting and repairing the function of endothelial cells and mitigating endothelial cell injury is of vital importance in the prevention and treatment of AS.

Atorvastatin (ATV) is a widely prescribed medication used to treat hypercholesterolemia and reduce cholesterol and triglyceride levels. It is an HMG-CoA reductase inhibitor and has been demonstrated to be effective in primary and secondary prevention studies of cardiovascular diseases. ATV significantly reduces the risk of cardiovascular events, with a 1 mmol/L reduction in low-density lipoprotein (LDL) cholesterol resulting in a 22% decrease in cardiovascular events [6]. ATV is a commonly used drug for the treatment of AS. It has been shown to effectively lower levels of LDL cholesterol and improve cardiovascular outcomes in both diabetic and nondiabetic patients [7]. Marquardt et al. demonstrated a significant reduction in the prevalence of carotid stenosis (a disease associated with AS) with the use of ATV [8]. The current understanding is that ATV alleviates AS primarily by inhibiting the enzymatic activity of HMG-CoA reductase in the liver, thereby reducing cholesterol synthesis [9]. On the other hand, it may also reduce the infiltration of inflammatory cells, the release of inflammatory mediators, and the production of inflammatory factors, thereby alleviating the inflammatory response within the plaque [10]. However, the precise molecular mechanisms of its therapeutic effects require further investigation.

Ferroptosis is an iron-dependent programmed cell death characterized by the accumulation of lipid peroxides and impaired glutathione (GSH) redox activity. Excessive intracellular free iron triggers iron deposition, resulting in lipid (hydro) peroxidation and subsequent cell death. It differs morphologically, biochemically, and genetically from other forms of cell death, such as apoptosis, necrosis, and autophagy [11–13]. Iron-mediated cell death has been found to promote the development of AS by accelerating endothelial dysfunction in lipid peroxidation [14]. Furthermore, dysregulation of intracellular iron impairs macrophages, vascular smooth muscle cells, and endothelial cells, affecting many risk factors or pathological processes of AS, such as lipid peroxidation, oxidative stress, inflammation, and dyslipidemia [14]. Macrophages have been found to be influenced by iron overload and iron deposition, contributing to the progression of AS. Additionally, dietary iron overload and elevated nontransferrin-bound iron (NTBI) have been identified as risk factors for AS, further supporting the link between iron and AS [15, 16]. Therefore, targeting ferroptosis may offer a new direction for the treatment of AS.

It has been found that ATV alleviates iron-dependent myocardial injury and inflammation following coronary microembolization through the Hif1a/Ptgs2 signaling pathway [17]. ATV can suppress iron-dependent cell death in H9c2 cells by regulating SMAD7/Hepcidin expression, thereby improving myocardial ischemia-reperfusion injury [18]. On the other hand, acyl-CoA synthetase long-chain family member 4 (ACSL4) can acylate polyunsaturated fatty acids to influence ferroptosis and serves as a marker of sensitivity to iron-dependent cell death, which can be modulated [19]. Therefore, to investigate whether ATV mitigates AS by modulating ACSL4-mediated ferroptosis, we applied an in vitro model [20] of AS using oxidized LDL (ox-LDL)-induced human umbilical vein endothelial cells (HUVECs) to elucidate the potential molecular mechanisms of ATV.

## 2. Materials and Methods

### 2.1. HUVEC Treatment

HUVECs, provided by Procell Co., Ltd. (Wuhan, China), were cultured in a specific medium consisting of Ham's F-12 K supplemented with 0.1 mg/mL heparin, 0.05 mg/mL endothelial cell growth supplement, and 10% fetal bovine serum. The cell culture was maintained in a 37°C incubator with 5% CO_2_.

Ox-LDL was purchased from Yeasen (Shanghai, China), while ATV was obtained from Solarbio (Beijing, China). In order to establish endothelial cell injury models for AS, HUVECs were treated with various concentrations of ox-LDL (0, 25, 50, 100, and 200 mg/L). ATV was dissolved in 5% ethanol at a concentration of 20 mg/mL, and the ox-LDL-treated HUVECs were subsequently treated with different concentrations of ATV (0, 2.5, 5, and 10 *μ*M) for a period of 24 h. The ethanol concentration in each group was adjusted to match that of the 10 *μ*M group. Subsequent experiments were performed using ox-LDL at 100 mg/L and ATV at 10 *μ*M.

### 2.2. Cell Viability Assay

HUVECs (1 × 10^4^ cells per well) were seeded in 96-well plates (Nest Biotechnology) and incubated with the specified concentrations of chemicals for a duration of 12 h. After the treatment period, 10 *μ*L of CCK-8 reagent was added to each well and incubated at 37°C for 2 h. The optical density (OD) at 450 nm was measured using a microplate reader to assess cell viability. Each experimental group was performed in triplicate (*n* = 3).

### 2.3. Measurement of Lactate Dehydrogenase (LDH)

Cellular cytotoxicity was evaluated by measuring the release into the culture medium. The culture medium was collected, and cell-free supernatants were obtained. The activity of LDH in the medium was measured using a commercially available LDH assay kit (Thermo Fisher Scientific, USA), following the instructions provided by the manufacturer. Each experimental group was performed in triplicate (*n* = 3).

### 2.4. Cell Apoptosis Assay

The Annexin V-FITC Apoptosis Detection Kit (Invitrogen) was utilized for the apoptosis assay. Cells were washed with PBS and then resuspended in 200 *μ*L of binding buffer at a density of 3 × 10^5^ cells/mL. Subsequently, 5 *μ*L of Annexin V-FITC and 10 *μ*L of propidium iodide (PI) were added to the cell suspension. The analysis of cell apoptosis was performed using a FACS cytometer (BD Biosciences, San Jose, CA, USA). Each experimental group was performed in triplicate (*n* = 3).

### 2.5. ELISA

Protein secretions of the typically released cytokines in AS were measured using ELISA kits for TNF-*α*, IL-6, and IL-8 according to the manufacturer's instructions (Abcam, USA). The absorbance was measured at 450 nm using a microplate reader. Each experimental group was performed in triplicate (*n* = 3).

### 2.6. Iron Assay

Intracellular ferrous ion levels were detected using an Iron Content Test Kit (ab83366, Abcam, Cambridge, UK). According to the manufacturer's instructions, the cells were lysed, the supernatant was collected by centrifugation, and the iron content was determined according to the instructions. The absorbance was measured at 560 nm. Each experimental group was performed in triplicate (*n* = 3).

### 2.7. Lipid Peroxidation Assay

The concentration of malondialdehyde (MDA) in cells was determined using the MDA kit from Abcam. HUVECs were harvested and subjected to repeated freeze-thaw cycles until complete lysis was achieved. The resulting cell lysate was then centrifuged at 4°C and 10,000 g for 10 min, and the supernatant was collected. The absorbance of the supernatant was measured following the instructions provided with the kit. Each experimental group was performed in triplicate (*n* = 3).

### 2.8. GSH Assay

The activity of reduced GSH was detected using the GSH kit (Abcam). HUVECs were harvested and added to the extraction reagent in the kit. After centrifugation, absorbance values were measured according to the kit instructions. Each experimental group was performed in triplicate (*n* = 3).

### 2.9. Lipid Reactive Oxygen Species (ROS)

Intracellular lipid ROS was analyzed by flow cytometry. The cells were treated and incubated in a medium with 2 mM BODIPY 581/591C11 dye (Invitrogen) at 37°C for 20 min. The fluorescence intensity of BODIPY 581/591C11-stained cells was measured by flow cytometry. Each experimental group was performed in triplicate (*n* = 3).

### 2.10. Cell Transfection

Overexpression of ACSL4 (oe-ACSL4) and its negative control OE (oe-NC) were provided by GenePharma, Shanghai. Cells were transfected with oe-ACSL4 or oe-NC using Lipofectamine 2000 (Invitrogen) for 48 h.

### 2.11. Immunofluorescence (IF) Assay

For the IF assay, cell slides were placed into a 12-well plate, with one slide per well, and HUVECs were cultured on these slides. The treated cells were fixed with 4% formaldehyde containing 0.25% Triton 100 for 20 min. After blocking, the cells were incubated with specific primary antibodies against ACSL4 (1:50, 22401-1-AP, Proteintech, USA) at room temperature (RT) for 2 h, followed by incubation with secondary antibodies for 40 min. DAPI was used as a counterstain for cell nuclei, and the cells were visualized using a microscope (BA410T, Motic, China). Each experimental group was performed in triplicate (*n* = 10).

### 2.12. Reverse Transcription-Quantitative Polymerase Chain Reaction (RT-qPCR)

The HUVECs in each group were treated and suspended in TRIzol reagent (Invitrogen). Total RNA was extracted and converted into cDNA using a PrimeScript™ RT reagent kit with a gDNA eraser (Takara, Otsu, Japan). SYBR Premix Ex Taq II (Takara, Otsu, Japan) was then used for qPCR amplification of cDNA. The 2^−ΔΔCT^ method was used to analyze the relative fold change.

### 2.13. Western Blot

For the Western blot analysis, after the specified treatment, the cells were lysed using a cell lysis buffer supplemented with protease and phosphatase inhibitors. The proteins were extracted and denatured by heating at 100°C for 5 min. Equal amounts of protein samples were separated by 10% sodium dodecyl sulfate-polyacrylamide gel electrophoresis (SDS-PAGE) and transferred onto polyvinylidene difluoride (PVDF) membranes (Bio-Rad, USA). The membranes were then blocked with a 5% nonfat milk-blocking solution in Tris-buffered saline containing 0.01% Tween 20 (TBST). Subsequently, the membranes were incubated overnight at 4°C with specific primary antibodies, followed by incubation with HRP-conjugated secondary antibodies for 2 h at RT. The protein bands were visualized using an enhanced chemiluminescence (ECL) kit (Thermo Fisher Scientific, USA). Each experimental group was performed in triplicate (*n* = 3).

### 2.14. Statistical Analysis

Statistical analysis was performed using GraphPad Prism 8 (GraphPad, La Jolla, CA, USA). Statistical significance was assessed using Student's *t*-test or analysis of variance (ANOVA) test between two groups or among multiple groups. All data were obtained from three independent repetitions and presented as mean ± standard deviation (SD). ^∗∗^*p* < 0.05 indicated statistical significance.

## 3. Results

### 3.1. ATV Alleviated Ox-LDL-Induced HUVEC Damage

**Figure 1:**
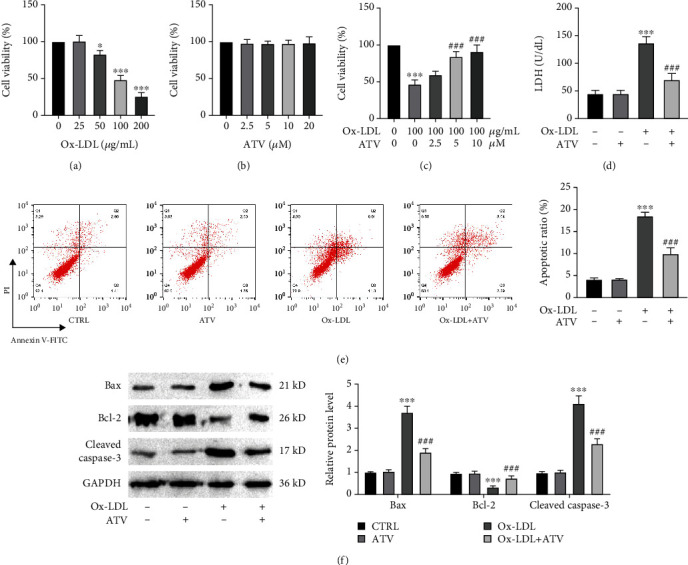
ATV alleviates ox-LDL-induced HUVEC damage. (a) Cell viability significantly decreased in a dose-dependent manner following ox-LDL treatment (CCK-8 assay). (b) Different concentrations of ATV have no effect on cell viability (CCK-8 assay). (c) ATV significantly improved the loss of cell viability induced by ox-LDL (CCK-8 assay). (d) ATV inhibited ox-LDL-induced LDH release from cells (LDH assay). (e, f) ATV suppressed ox-LDL-induced cell apoptosis (flow cytometry and Western blot). ATV, atorvastatin; ox-LDL, oxidized low-density lipoprotein. ^∗^*p* < 0.05 and ^∗∗∗^*p* < 0.001: compared to the CTRL group; ^##^*p* < 0.01 and ^###^*p* < 0.001: compared to the ox-LDL group.

HUVECs were subjected to ox-LDL-induced damage, and it was found that the cell viability was reduced to 50% compared to the control group when the ox-LDL concentration was 100 *μ*g/mL, which was subsequently used for all experiments (Figure 1(a)). Different concentrations of ATV have no effect on cell viability (Figure 1(b)), while the addition of different concentrations of ATV restored cell viability after ox-LDL treatment, and significant improvements were observed at ATV concentrations of 5 and 10 *μ*M (Figure 1(c)). Following ox-LDL treatment, the LDH levels in the cells significantly increased, while the addition of ATV significantly mitigated the elevation of LDH levels (Figure 1(d)). Flow cytometry analysis comparing the levels of apoptosis in the control group, ox-LDL group, and ox-LDL + ATV group revealed that ox-LDL significantly increased the proportion of apoptotic cells, while ATV treatment reduced ox-LDL-induced cell apoptosis (Figure 1(e)). Through analysis of the expression of apoptotic proteins Bax, cleaved caspase-3, and antiapoptotic protein Bcl-2, it was found that ox-LDL significantly increased the protein levels of Bax and cleaved caspase-3 and decreased the protein level of Bcl-2. However, ATV effectively mitigated the elevated levels of Bax and cleaved caspase-3 induced by ox-LDL while concurrently enhancing the depleted protein expression of Bcl-2 caused by ox-LDL (Figure 1(f)).

### 3.2. ATV Attenuated Ox-LDL-Induced Inflammation and Adhesion in HUVECs

**Figure 2:**
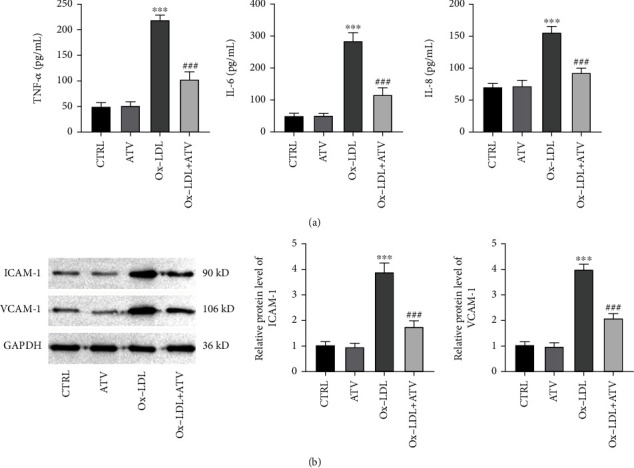
ATV alleviates ox-LDL-induced inflammation and adhesion in HUVEC. (a) ATV inhibited the release of inflammatory cytokines induced by ox-LDL (ELISA). (b) ATV suppressed the increased protein expression of ICAM-1 and VCAM-1 induced by ox-LDL (Western blot). ATV, atorvastatin; ox-LDL, oxidized low-density lipoprotein. ^∗∗^*p* < 0.01 and ^∗∗∗^*p* < 0.001: compared to the CTRL group; ^#^*p* < 0.05, ^##^*p* < 0.01, and ^###^*p* < 0.001: compared to the ox-LDL group.

ELISA assays revealed that ox-LDL significantly increased the levels of inflammatory factors TNF-*α*, IL-6, and IL-8 in HUVECs, while ATV treatment significantly reduced the elevation of inflammatory factor levels induced by ox-LDL (Figure 2(a)). Detection of the protein expression levels of cell adhesion molecules ICAM-1 and VCAM-1 revealed that ox-LDL significantly increased the levels of adhesion molecules in HUVECs, while ATV treatment significantly decreased the levels of adhesion molecules induced by ox-LDL (Figure 2(b)).

### 3.3. ATV Inhibited Ferroptosis in Ox-LDL-Induced HUVECs

**Figure 3:**
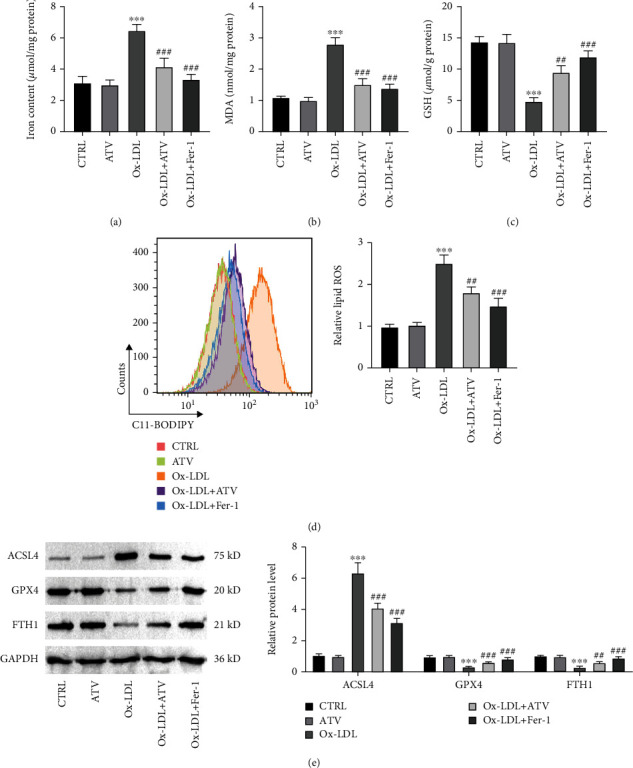
ATV inhibits ferroptosis in ox-LDL-induced HUVEC. (a–d) Compared to the control group, ox-LDL treatment resulted in decreased GSH levels and increased iron content, MDA, and lipid ROS levels in HUVEC. However, treatment with ATV or Fer-1 promoted GSH levels and inhibited iron content, MDA, and lipid ROS levels induced by ox-LDL. (e) Compared to the control group, ox-LDL treatment led to increased expression of ACSL4 and decreased expression of GPX4 and FTH1 in HUVEC. However, treatment with ATV or Fer-1 suppressed ACSL4 protein expression and promoted the expression of GPX4 and FTH1 in ox-LDL-induced HUVEC (Western blot). ATV, atorvastatin; ox-LDL, oxidized low-density lipoprotein. ^∗∗∗^*p* < 0.001: compared to the CTRL group; ^#^*p* < 0.05, ^##^*p* < 0.01, and ^###^*p* < 0.001: compared to the ox-LDL group.

To evaluate whether ATV regulates ferroptosis in HUVECs, we first observed the phenotypes related to ferroptosis. It was found that ox-LDL induction significantly increased the cellular iron content, MDA levels, ROS levels, and ACSL4 protein levels. On the other hand, the levels of ferritin heavy chain 1 (FTH1), the antioxidant GSH, and the GSH peroxidase 4 (GPX4) protein were significantly reduced (Figures 3(a), 3(b), 3(c), 3(d), and 3(e)). Treatment with ATV or the iron inhibitor Fer-1 significantly reduced the cellular iron content, MDA levels, ROS levels, and ACSL4 protein levels induced by ox-LDL. Conversely, the levels of GSH, GPX4, and FTH1 protein were significantly increased (Figure 3(a), 3(b), 3(c), 3(d), and 3(e)).

### 3.4. ATV Protected HUVECs From Ferroptosis by Inhibiting ACSL4

**Figure 4:**
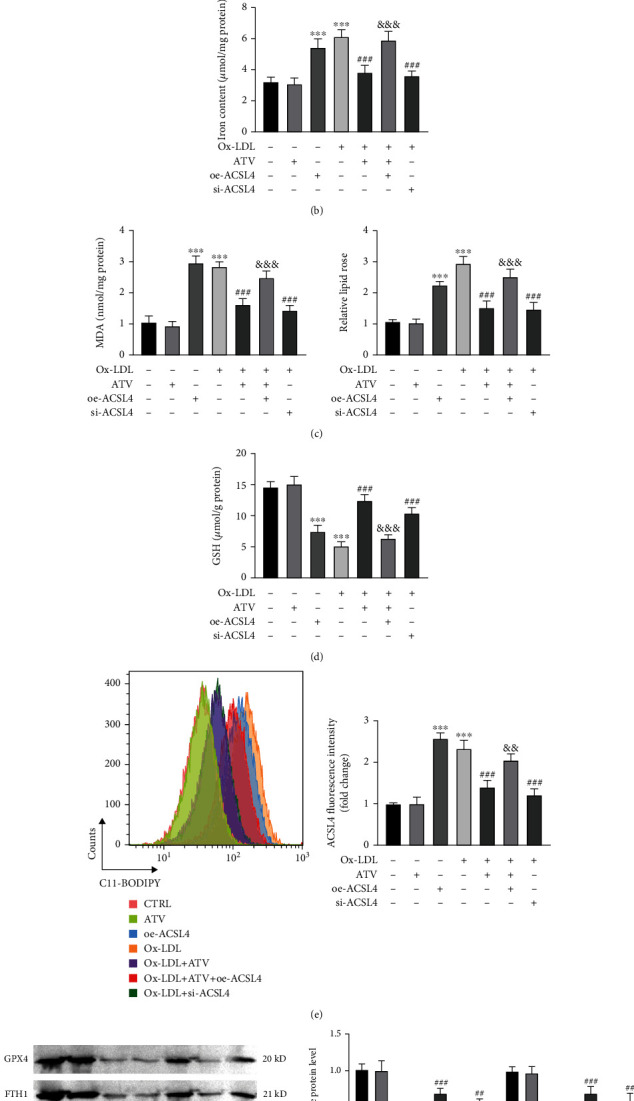
ATV protects HUVECs from ferroptosis by suppressing ACSL4. (a) Overexpression of ACSL4 in HUVECs treated with ATV (immunofluorescence). (b, c) Overexpression of ACSL4 reversed the inhibitory effect of ATV on intracellular iron and MDA levels in ox-LDL-induced HUVEC. (d) Overexpression of ACSL4 attenuated the promoting effect of ATV on GSH levels in ox-LDL-induced HUVECs. (e) The inhibitory effect of ATV on lipid ROS levels in ox-LDL-induced HUVECs was weakened by overexpression of ACSL4. (f) Overexpression of ACSL4 inhibited the promoting effect of ATV on the protein expression of GPX4 and FTH1 in ox-LDL-induced HUVECs. ATV, atorvastatin; oe-ACSL4, overexpression of ACSL4; ox-LDL, oxidized low-density lipoprotein. ^∗∗∗^*p* < 0.001: compared to the CTRL group; ^##^*p* < 0.01 and ^###^*p* < 0.001: compared to the ox-LDL group; ^&^*p* < 0.05, ^&&^*p* < 0.01, and ^&&&^*p* < 0.001: compared to the ox-LDL + oe-NC group.

To further evaluate whether ATV inhibits cell ferroptosis by downregulating ACSL4 expression, we overexpressed ACSL4 in the cells (oe-ACSL4) and found that oe-ACSL4 treatment significantly increased the levels of ACSL4 (Figure 4(a)), which hindered the downregulation of cellular iron levels (Figure 4(b)), MDA levels (Figure 4(c)), and ROS levels (Figure 4(e)). On the other hand, it was observed that oe-ACSL4 hindered the upregulation of GSH levels induced by ATV under ox-LDL induction (Figure 4(d)), as well as the expression of GPX4 protein and FTH1 protein (Figure 4(f)).

### 3.5. ATV Alleviated Ox-LDL-Induced HUVEC Damage by Inhibiting ACSL4-Mediated Ferroptosis

**Figure 5:**
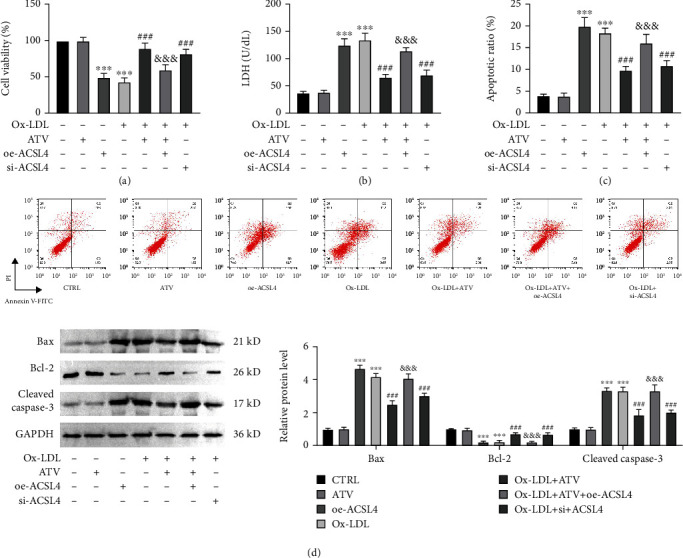
ATV alleviates ox-LDL-induced HUVEC damage by suppressing ACSL4-mediated ferroptosis. (a) The promoting effect of ATV on cell viability in ox-LDL-induced HUVECs was attenuated by overexpression of ACSL4 (CCK-8 assay). (b) Overexpression of ACSL4 reversed the inhibitory effect of ATV on LDH levels in ox-LDL-induced HUVECs (LDH assay). (c, d) The inhibitory effect of ATV on apoptosis in ox-LDL-induced HUVECs was weakened by overexpression of ACSL4. ATV, atorvastatin; oe-ACSL4, overexpression of ACSL4; ox-LDL, oxidized low-density lipoprotein. ^∗∗∗^*p* < 0.001: compared to the CTRL group; ^##^*p* < 0.01 and ^###^*p* < 0.001: compared to the ox-LDL group; ^&^*p* < 0.05, ^&&^*p* < 0.01, and ^&&&^*p* < 0.001: compared to the ox-LDL + oe-NC group.

Subsequently, we observed cell damage in ACSL4-overexpressing cells. We found that oe-ACSL4 hindered the protective effects of ATV on cell viability (Figure 5(a)) and the upregulation of the antiapoptotic protein Bcl-2 (Figure 5(d)). Additionally, oe-ACSL4 attenuated the downregulation of LDH levels (Figure 5(b)), the proportion of apoptotic cells (Figure 5(c)), and the expression levels of proapoptotic proteins Bax and cleaved caspase-3 (Figure 5(d)) induced by ox-LDL.

### 3.6. ATV Alleviated Ox-LDL-Induced Inflammation and Adhesion in HUVECs by Inhibiting ACSL4-Mediated Ferroptosis

**Figure 6:**
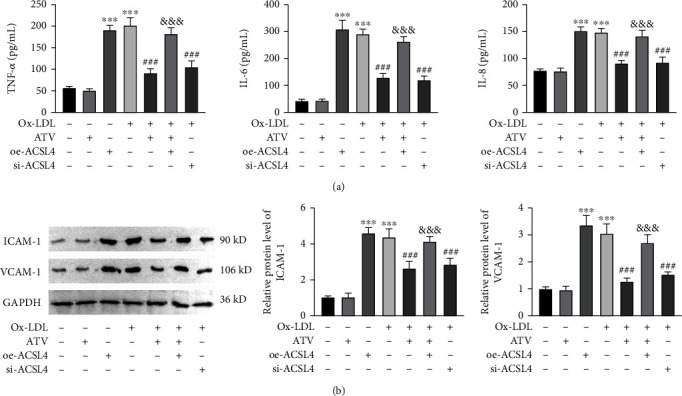
ATV attenuates inflammation and adhesion in ox-LDL-induced HUVECs by suppressing ACSL4-mediated ferroptosis. (a) In ox-LDL-induced HUVECs, overexpression of ACSL4 reversed the inhibitory effect of ATV on cellular inflammatory response (ELISA). (b) The inhibitory effect of ATV on the protein expression of ICAM-1 and VCAM-1 in ox-LDL-induced HUVECs was weakened by overexpression of ACSL4 (Western blot). ATV, atorvastatin; oe-ACSL4, overexpression of ACSL4; ox-LDL, oxidized low-density lipoprotein. ^∗∗∗^*p* < 0.001: compared to the CTRL group; ^##^*p* < 0.01 and ^###^*p* < 0.001: compared to the ox-LDL group; ^&&^*p* < 0.01 and ^&&&^*p* < 0.001: compared to the ox-LDL + oe-NC group.

Furthermore, upon observation of the levels of inflammatory factors and adhesion molecules, we observed that oe-ACSL4 hindered the downregulation of inflammatory factors TNF-*α*, IL-6, and IL-8 induced by ATV under ox-LDL induction (Figure 6(a)). Similarly, the downregulation of adhesion molecules ICAM-1 and VCAM-1 protein levels induced by ATV was also hindered by oe-ACSL4 (Figure 6(b)). These findings suggest that oe-ACSL4 attenuates the inhibitory effects of ATV on the levels of inflammatory factors and adhesion molecules in HUVECs under ox-LDL-induced conditions.

## 4. Discussion

LDH is an intracellular enzyme involved in cellular energy metabolism. During the development of AS, arterial endothelial cells are subjected to factors such as oxidative stress, inflammation, and ischemia, leading to cellular damage. LDH is released into the extracellular space during cell injury and necrosis, suggesting that LDH levels may increase in AS [21]. We also observed a significant increase in LDH levels in ox-LDL-induced HUVECs, indicating cellular damage induced by ox-LDL. However, treatment with ATV partially inhibited the increase in LDH levels and alleviated the ox-LDL-induced cellular membrane damage.

Cell apoptosis plays a crucial role in the development of AS, affecting plaque stability and arterial function. In the early stages of AS, endothelial cells subjected to inflammatory stimulation and injury may undergo apoptosis [22]. Endothelial cell apoptosis leads to impaired endothelial function, further promoting inflammation and plaque formation. Bax is a proapoptotic protein that increases mitochondrial outer membrane permeability, leading to the release of cytochrome c and activating the apoptotic pathway [23]. Bcl-2 is an antiapoptotic protein that inhibits the activity of Bax, maintains mitochondrial membrane integrity, and prevents apoptosis from occurring [24]. Caspase-3 is a key enzyme in the process of cell apoptosis. Cleaved caspase-3 (activated caspase-3) is the active form formed by the cleavage activation of procaspase-3 [25]. Detection or increased expression of cleaved caspase-3 is often used as a marker of cell apoptosis, indicating the activation of the apoptotic signaling pathway. In this study, ox-LDL induction increased the proportion of apoptotic HUVECs, accompanied by increased levels of proapoptotic protein Bax and cleaved caspase-3 and decreased levels of antiapoptotic protein Bcl-2, indicating ox-LDL-induced apoptosis of HUVECs and resultant cellular membrane damage. Treatment with ATV reduced ox-LDL-induced cell apoptosis and exerted a protective effect.

In the early stages of AS, damaged endothelial cells release inflammatory mediators such as cytokines and chemokines. These inflammatory mediators can upregulate the expression of cell adhesion molecules on the vascular endothelium, such as selectins, integrins, and adhesion molecules [26, 27]. This upregulation increases the adhesion of leukocytes to endothelial cells, allowing leukocytes to enter the arterial wall. Accumulated leukocytes within the arterial intima transform into macrophages, engulf ox-LDL, and form foam cells. These foam cells, along with other cells, contribute to the formation of plaques, which obstruct the arteries. Our study found that ox-LDL induced the expression of adhesion molecules ICAM-1 and VCAM-1 in HUVECs, while ATV was able to inhibit the increase in ICAM-1 and VCAM-1 protein levels. However, oe-ACSL4 hindered the inhibitory effect of ATV on the increase in ICAM-1 and VCAM-1 protein levels. This suggests that ATV mitigates ox-LDL-induced inflammation and adhesion in HUVECs by inhibiting ACSL4. These findings demonstrate the potential of ATV in ameliorating cellular damage, apoptosis, and adhesion induced by ox-LDL in the context of AS.

Ferroptosis is a newly discovered form of cell death characterized by the accumulation of intracellular labile iron and oxidative stress. In this process, labile iron catalyzes the production of highly reactive oxygen radicals, leading to lipid peroxidation and cellular membrane damage, ultimately resulting in cell death [28]. MDA and GSH are important molecular markers associated with oxidative stress and cellular damage. During ferroptosis, the increased intracellular labile iron and oxidative stress exacerbate lipid peroxidation, leading to the oxidation of unsaturated fatty acids in lipids and the generation of reaction products such as MDA. Therefore, MDA levels are often used as indicators of oxidative stress and ferroptosis [29]. GSH, a reduced form of the tripeptide GSH (glutamyl–cysteinyl–glycine), functions as an antioxidant. It plays a crucial role in cellular antioxidant defense and detoxification by aiding in the clearance of free radicals and harmful substances. In the process of ferroptosis, oxidative stress leads to increased consumption of intracellular GSH, disrupting the cellular antioxidant balance. Therefore, GSH levels are commonly associated with oxidative stress and ferroptosis [30]. ACSL4 is an acyl-CoA synthetase involved in fatty acid metabolism and synthesis within cells, particularly in phosphatidylinositol (PI) metabolism and inflammatory responses. Pathological processes such as inflammation and oxidative stress in AS may stimulate the expression of ACSL4. It has been found that the expression level of ACSL4 is often upregulated under conditions of ferroptosis [31]. The fatty acylation catalyzed by ACSL4 leads to an increase in the phosphatidylethanolamine (PE) content on cell membranes, making the membranes more susceptible to lipid peroxidation and promoting ferroptosis. Additionally, ACSL4 interacts with GPX4, a key enzyme involved in regulating intracellular GSH levels [19]. GPX4 is an important antioxidant enzyme that protects cells from oxidative stress damage by reducing lipid peroxidation products. The fatty acylation reaction catalyzed by ACSL4 consumes GSH and reduces the reductive capacity of GPX4, thereby increasing cellular sensitivity to ferroptosis. The accumulation of intracellular labile iron is considered a critical trigger in the mechanism of ferroptosis. Labile iron can catalyze the production of highly reactive oxygen radicals, initiating lipid peroxidation and cellular membrane damage, ultimately leading to cell death. The FTH1 gene encodes ferritin, a major intracellular iron storage protein in prokaryotes and eukaryotes. When cells require iron ions, FTH1 can dissociate and release iron ions to meet cellular demands. Conversely, when intracellular iron levels are excessive or under oxidative stress, FTH1 can increase iron storage to maintain iron ion balance [32]. By upregulating the expression of FTH1, cells can regulate iron metabolism and reduce the concentration of labile iron, thereby decreasing their sensitivity to intracellular iron-induced cell death. The presence of FTH1 provides a protective mechanism to prevent excessive labile iron from triggering ferroptosis. In isoproterenol-induced heart failure, FTH1 protein levels are downregulated, while treatment with ATV impedes the downregulation of FTH1 [33].

Our study revealed that in ox-LDL-induced cellular membrane damage, there is an increase in iron content, MDA levels, ACSL4 expression, and ROS levels. Additionally, there is an increase in the proportion of apoptotic cells. The decrease in antioxidant factors such as GSH and GPX4 suggests that ox-LDL induces iron-dependent cell death, leading to cellular membrane damage. Furthermore, we observed an upregulation of inflammatory factors and adhesion molecules. Treatment with ATV alleviated cell apoptosis and oxidative stress, as well as reduced the levels of inflammatory factors and adhesion molecules, indicating that ATV mitigated cellular membrane damage. On the other hand, through oe-ACSL4 in our research group, we observed that ATV no longer alleviated ox-LDL-induced cellular membrane damage, indicating that ATV acts by inhibiting ACSL4 to reduce inflammation and adhesion in HUVECs.

The limitations of this study include the following: This study employed HUVECs to establish an in vitro model of AS. However, this model cannot fully replicate the complex physiological environment and the progression of AS in vivo. Therefore, the experimental results may not fully reflect the actual conditions of atherosclerotic disease. Moreover, the sample size of the study was relatively small because experiments were conducted using only one cell line. Further expansion of the sample size and validation of the reproducibility of the results can enhance the reliability and credibility of the study.

## Data Availability

The datasets generated and analyzed during the current study are available from the corresponding author on reasonable request.
